# Investigating the Nutritional and Recovery Habits of Tennis Players

**DOI:** 10.3390/nu10040443

**Published:** 2018-04-03

**Authors:** James A. Fleming, Robert J. Naughton, Liam D. Harper

**Affiliations:** 1 School of Human and Health Sciences, University of Huddersfield, Queensgate, Huddersfield HD1 3DH, UK; r.naughton@hud.ac.uk (R.J.N.); l.harper@hud.ac.uk (L.D.H.); 2 School of Sport Health and Applied Sciences, St Mary’s University, Twickenham TW1 4SX, UK

**Keywords:** carbohydrate, nutrition, performance, recovery, tennis

## Abstract

In this study, the nutritional and recovery habits of tennis players pre-, during, and post-match-play were investigated. Seventy tennis players completed a bespoke nutrition and recovery habits questionnaire, with questions related to the following areas: match preparation, intra-match nutritional habits, situation dependent variables, and post-match nutrition and recovery. On match day-1, the consumption of balanced meals consisting of carbohydrate (CHO), fat and protein, with some micronutrient considerations were reported by 51% of players. On match-days, CHOs were prioritised prior to match-play with CHO dominant meals consumed by the majority of players. During matches, all players adopted a nutritional strategy, with water (94%), banana(s) (86%) and sports drinks (50%) commonly used. Carbohydrate rich nutritional aids, including sports drinks (80%) and energy gels (26%) were utilised more readily during long matches (>2 h). The day after match-play, 39% of players reported the consumption of “nothing specific”. Multiple post-match recovery strategies were adopted by 80% of players, with foam rolling (77%), ice baths (40%), protein shake intake (37%) and hot baths (26%) most used. Findings indicate highly variable eating and recovery habits in tennis players pre-, during and post-match-play, with scope for improved practices.

## 1. Introduction

With an estimated 60 million players globally, tennis is considered one of the most popular sports in the world [1]. Tennis orientates around tournament play, which may result in a congested fixture calendar, frequent travel, and an unpredictable time spent in competitive match-play [2]. These factors subsequently pose several challenges that could influence a player’s preparation, match performance, and recovery.

Broadly considered an intermittent sport, tennis match play is characterized by rapid and explosive movements, including acceleration, deceleration, stretches, jumps and stroke-making from various body positions [3]. With match length varying from less than one hour to more than five hours, great mechanical and physiological stress is placed upon the body [4,5]. Performance variables, such as stroke velocity, running speed and maximum muscular force, have been reported to significantly decrease during a tennis match [6], with stroke accuracy decrements of 81% observed alongside increased play duration [7]. Gomes et al. [8] investigated the impact of one 3-h tennis match on recovery markers and found significant decrements in neuromuscular performance immediately post-match, and indirect markers of muscle damage (creatine kinase and serum myoglobin concentration) 24–48 h post-match. Furthermore, research has identified players’ physical performance and recovery to be compromised during multiple tennis matches as encountered in tennis tournaments [3]. Although rapid recovery is of critical importance to tennis performance [9], information relating to effective recovery strategy implementation for tennis is scarce [7]. Furthermore, what recovery strategies tennis players use following matches is not well known. Therefore, there is scope to assess the use of recovery strategies in tennis, to inform future studies that can investigate strategies’ efficacy and effectiveness.

An athlete’s nutritional intake has a direct influence on augmenting training adaptations [10], enhancing performance [11] and recovery [2], reducing risk of injury [12] and preventing illness [13]. Nutrition is considered a fundamental factor in the overall development of an athlete [1]. Understanding the food-related habits of sports people is important, as they influence energy consumption, nutrient intake and hydration status [14]. Several studies have investigated eating habits and nutritional knowledge in other sporting domains [15,16] yet very little has been carried out in tennis [2]. To date, only one study has investigated the nutritional profile of adolescent male tennis players [17]. Juzwiak et al. [17] reported an energy deficit of 32% and nutritional deficiencies in players, with carbohydrate (CHO) and micronutrient intakes concomitantly below recommended values [18], resulting in concerns regarding short and long-term impacts on growth, health and performance in this population. Although useful insight can be taken from this study, it failed to distinguish any tennis specific performance needs, a key consideration in the development of the athlete. Gaining an insight into the nutritional habits of athletes is important to enable practitioners to better direct, plan and implement appropriate nutrition education to enhance knowledge and potentially influence the dietary behaviours of athletes [15].

Therefore, the aims of the current study were twofold: (1) identify current player habits in relation to dietary intake pre-, during and post-match-play and (2) investigate recovery strategy use.

## 2. Materials and Methods

### 2.1. Participants and Study Design

Competitive tennis players were recruited from Europe and North America. Recruitment consisted of contacting players and coaches electronically, via telecommunications or in person between May and July 2017. Participants were recruited through professional and personal tennis links of the lead investigator. Furthermore, players and coaches were asked to inform other squad members and appropriate level players, and to pass on the lead investigator’s contact details. After initial contact, individuals were prompted after four weeks had elapsed in case they had not already participated in the study. From this recruitment strategy, 70 tennis players aged 19 ± 3 years participated. To be eligible for the study, participants had to be competing at county/provincial level or higher. Participants were asked to state their representative level, current club and tennis rating/ranking via their Lawn Tennis Association (LTA) rating, Universal Tennis Rating (UTR), and or current National/International Ranking. All participants gave informed consent, with parental consent gained for those under 18 years of age. Ethical permission was obtained from the University of Huddersfield Human and Health Sciences Ethics Committee (reference: SREP/2017/032, approval date: 8 May 2017).

Nutritional and recovery habits were assessed via a questionnaire (Supplementary Materials), as previously used in other sporting contexts [16,19]. The questionnaire was designed by two registered Sport and Exercise Nutritionists (SENr) and created using an online resource (Google Forms) with an approximate completion time of 10 min. Online data collection methods are deemed as viable methods, facilitating increased access to participants, and enhancing participant experience compared to paper-based methods [20]. Moreover, two independent researchers not involved in the design or execution of the study checked the face validity of the questionnaire prior to circulation. All responses were anonymous, with participants required to disclose their gender, age, competitive level, and tennis club location. The questionnaire contained a total of 28 items relating to four key topics, namely match preparation, intra-match nutrition, post-match nutrition and recovery, and situation-dependent variables. The questionnaire contained various questions in a scaled, rank or open-ended format. The unstructured or open-ended component allowed participants to provide further detail with regard to the nutritional and recovery strategies they use. The use of questionnaires that include open-ended questions has been recently recommended by Harper and McCunn [21] as the information gained can provide players, coaches, and nutritionists with specific details about the nutritional support players need. All questions were mandatory and had to be completed before the questionnaire could be submitted. Where ‘other’ was an option, participants were asked to elaborate. Participants completed the questionnaire online via a personal computer and/or mobile device with internet access. The questionnaire was accessible through individual case-protected web URL links, ensuring complete confidentiality.

### 2.2. Questionnaire Topics

#### 2.2.1. Match Preparation

Participants were asked to specify their pre-match habitual nutritional intake the day before, and day of competitive match-play. The impact of different start times (i.e., morning, midday, afternoon and evening) were assessed, with participants encouraged to elaborate on specifics relating to nutritional intake.

#### 2.2.2. Intra-Match Nutritional Habits

Participants specified what they normally eat and drink during a tennis match. Options included banana(s); energy gels; sweets; chocolate; Jaffa cakes (chocolate orange flavoured biscuit); water; sports drinks; juice/squash (fruit drink concentrate) and other.

Participants were asked how they manage fluid consumption during a match. The following options were given: number of games completed; at the change of ends; at the end of a set; at the end of a match; time passed; ad-libitum consumption according to thirst, and other. Participants were also asked if they specifically targeted a certain volume of fluid during a match (500 mL; 500 mL^−1^ L; >2 L; other).

#### 2.2.3. Situation Dependent Variables

Considerations made for match duration (>2 h) were examined with participants asked to provide actions taken (nutritional or otherwise) from the following options: gels; sports drinks; sweets; cold/wet towel and other. The potential influence of different playing surfaces (clay, tarmac/acrylic/hard, and grass) on nutritional intake were examined, with participants asked to specify any modifications to practice and to provide reasoning for their answers.

#### 2.2.4. Post-Match Nutrition and Recovery

Participants were asked to specify their habitual nutritional intake post competitive match-play. The impact of timing was assessed (i.e., immediately post, 3 h post, before bed, and the day after the match) with participants encouraged to elaborate on specifics relating to post match-play feeding/nutritional intake.

Recovery strategies habitually implemented were examined and participants were provided with the following options: ice bath; hot bath; compression garments; foam rolling; protein shakes; cherry juice; creatine and other. Participants were also asked to state the reason why they implement the recovery strategy/strategies chosen. Options included scientific literature; coach/peer influence; saves time; saves money; easily available; and personal preference.

### 2.3. Data Analysis

The study design is of a cross-sectional and descriptive nature and as such the data is presented in a descriptive manner. For questions with categorical responses, frequency and trends were determined. Written responses for open-ended questions were read several times to develop a full understanding of the content [22]. An inductive content analysis led by the lead author (J.A.F.) was then carried out following the organisation of the raw data. Themes were then established, and inductive analysis continued until data saturation had occurred. A sub-analysis was carried out to establish whether playing standard impacted nutritional habits and recovery practices. Twenty participants classified as ‘elite’ level players categorised by their Association of Tennis Professionals (ATP) ranking, International Tennis Federation (ITF) ranking, National ranking or UTR (>13)/LTA rating (<2.2) [23,24,25] were analysed against the remaining lower level players. Additional analyses of age were carried out: ‘junior’ (<18 years old, *n* = 15) and ‘adult’ players (18–35 years old, *n* = 55), and gender (male *n* = 48, female, *n* = 22).

## 3. Results

### 3.1. Match Preparation

When asked what players eat the day before competitive match-play, 51% (*n* = 36) opt for balanced meals (consisting of CHOs, fats and protein and some micronutrient considerations). Twenty-seven percent (*n* = 19) choose CHO dominant meals and 13% (*n* = 9) select CHOs and protein sources only to form their match day (MD)-1 nutrition regimes. Results regarding nutritional habits on MD indicate CHO dominant meals as the favoured food choice. Fifty percent (*n* = 35) opt for CHO dominant meals prior to a morning match, 43% (*n* = 30) prior to a mid-day match, 63% (*n* = 44) before an afternoon match and 56% (*n* = 39) prior to an evening match.

From the 51% of players who stated they consume balanced meals on MD-1, just 11% (*n* = 8) opt for them on the day of a morning match. Additionally, only 20% (*n* = 14) reported eating balanced meals prior to a mid-day match, 16% (*n* = 11) prior to an afternoon match and 13% (*n* = 9) before an evening match. The consumption of similar foods was reported alongside morning match preparation, with 41% (*n* = 29) eating oatmeal/cereals, and 31% (*n* = 22) eating eggs. Prior to an afternoon match, CHO rich foods, including pasta (46%, *n* = 32) and sandwiches (36%, *n* = 25), were regular food choices. Similar habits were noted prior to an evening match, with 51% (*n* = 36) and 23% (*n* = 16) consuming pasta and sandwiches respectively. The following two themes emerged: ‘pre-match meal focus’ (e.g., “Usually only concentrate on dinner [before an evening match]. Dinner consists of carbs, veg and chicken. Sometimes rice and sometimes pasta”), and digestibility (e.g., “…Preferably whole grain pasta or brown rice as this is slower releasing. Nothing that takes too long to digest”).

### 3.2. Intra-Match Nutritional Habits

**Figure 1:**
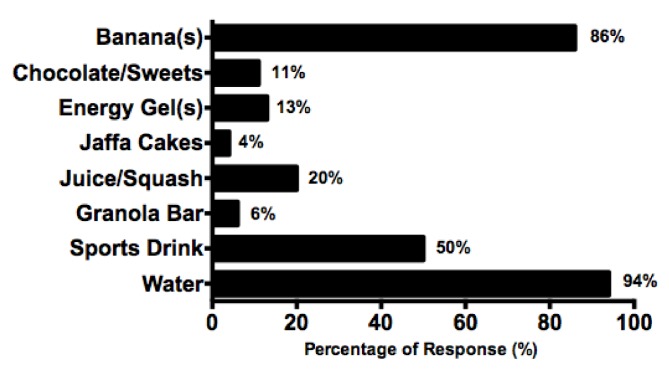
Match-Play nutrition consumption, expressed as percentages. Other responses included the consumption of figs, dates, branched chain amino acids (BCAAs), a cube of jelly, and homemade fruit cupcakes; all cited once by different players.

Water (94%, *n* = 66) and banana consumption (86%, *n* = 60) were the most cited nutritional considerations during match-play (Figure 1). Ninety-four percent (*n* = 66) of players also reported the consumption of multiple food/fluid items during match-play, with the majority (70%, *n* = 49) consuming three.

When asked how they gauge fluid demands during match-play 69% (*n* = 48) of players reported doing so at the change of ends, with 29% (*n* = 20) reporting ab-libitum consumption throughout the match. Themes emerged regarding game/match intensity (e.g., “certain games may be longer and tougher so you may need to consume more fluids at the change of ends to account for this”) and weather/heat considerations (e.g., “if I’m playing in the heat I will try to drink more at the change of ends to keep hydrated and replace fluids that have been lost”). In terms of targeting a specific volume of fluid during match-play, only 23% (*n* = 16) of players reported doing so; with eight targeting 500 mL^−1^ L, one 1–2 L and seven >2 L.

### 3.3. Situation Dependent Variables

**Figure 2:**
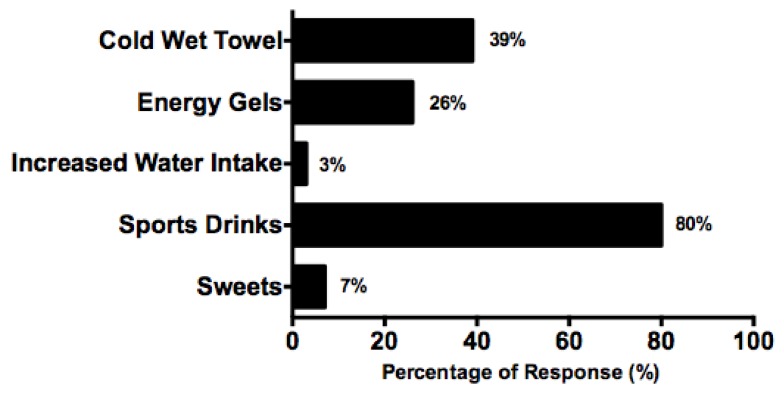
Considerations made by players during a long match (>2 h), expressed as a percentage.

When asked what considerations are made during a long match (>2 h) players reported the additional consumption of sports drinks (80%, *n* = 56), energy gels (26%, *n* = 18) and sweets (7%, *n* = 5). Thirty-nine percent (*n* = 27) reported using cold/wet towels during breaks in play (Figure 2). Fifty nine percent (*n* = 41) of players opt for multiple performance aids.

Fourteen percent (*n* = 10) of players reported changing their eating habits when playing on clay courts (e.g., “eating more”). No changes were reported when asked if they changed their eating habits on hard/acrylic/tarmac or grass courts.

### 3.4. Post-Match Nutrition and Recovery

Results indicated varied eating habits immediately post-match with 34% (*n* = 24) of players opting for CHO dominant meals, 26% (*n* = 18) have protein shakes, 19% (*n* = 13) have balanced meals, 9% (*n* = 6) have sports drinks, and 9% consume nothing at all (*n* = 6). Meal consumption within 3-h’ post-match shifted, with 61% of players (*n* = 43) reporting the consumption of a balanced meal. Before bed, post-match nutrition habits highlighted some consideration of smaller portions (*n* = 4), with the largest proportion of players (44%, *n* = 31) reporting consumption of a balanced meal. When asked what they typically eat or drank the day after matches for recovery, 39% (*n* = 27) reported the consumption of nothing specific. Two themes emerged regarding post-match nutrition habits: availability (e.g., “if travelling then it would be whatever is available fast food wise”), and practicality (e.g., “availability at the venue”, “wherever the team dines”).

**Figure 3:**
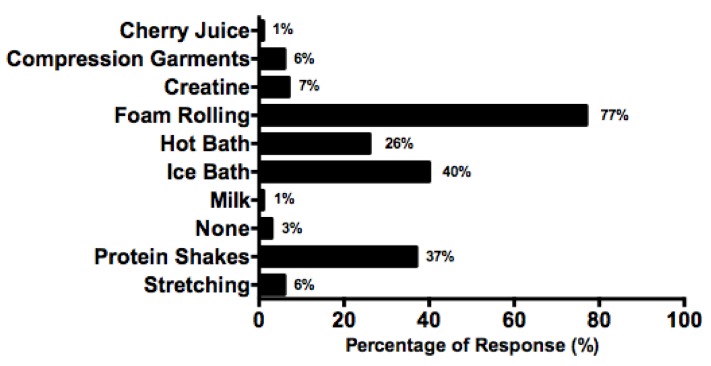
Post-match recovery strategy use, expressed as a percentage.

Foam rolling was the most commonly cited method, followed by ice baths, protein shakes and hot baths (Figure 3). Eighty percent of players (*n* = 56) recorded utilising multiple recovery strategies with the four aforementioned recovery strategies cited most. Additionally, two players cited using ‘other’ recovery strategies which included beetroot juice and homemade salt mineral solution. Two players noted having no recovery strategy.

When asked why they used recovery strategies, a range of reasons were given. ‘Coach/Peer influence’ was the main reason provided for adopting foam rolling (56%) and ice bath (43%) use. Scientific literature was the second most cited reason given, accounting for 17% for foam rolling, 36% for ice baths and 31% for protein shake use (Table 1).

### 3.5. Sub-Analysis by Playing Standard–‘Elite’ vs. Lower Level Players

No clear differences were observed between the two cohorts with the exception of nutrition habits surrounding match preparation. Sixty percent of ‘elite players’ opt for CHO dominant meals as part of their morning match preparation compared to 49% of the lower level players. Nutritional habits prior to afternoon and evening matches indicated that 80% and 75% of the elite cohort focus on CHO dominant meals compared to 64% and 55% of the lower level players, respectively.

### 3.6. Sub-Analysis by Age–Juniors vs. Adult Players

Differences were observed between junior and adult players regarding eating habits and match preparation. Eighty percent of junior players (>18 years old) chose CHO dominant meals alongside their pre-match meal consumption, compared to 44% of adult players (18–35 years old), with no notable differences observed in any other areas.

### 3.7. Sub-Analysis by Gender

No notable differences were observed between gender, nutritional and recovery habits.

## 4. Discussion

Aligning with the aims of the study, a notable shift towards increased CHO intake on MD was observed. Sixty-three percent and 56% of players opt for CHO dominant meals alongside their pre-match routine when partaking in an afternoon and or evening match, respectively (up from 27% on MD-1), demonstrating some recognition of the importance of CHOs prior to matches. There is significant evidence supporting ergogenic effects of CHO consumption prior to exercise on physical performance [26,27]. The performance of sustained or intermittent high-intensity exercise is enhanced by nutrition strategies that maintain high CHO availability [11]. Failure to meet these demands has been associated with fatigue in the form of reduced work rates, impaired skill and concentration, and increased perception of effort [3,27].

A sub-analysis highlighted a notable increase in CHO dominant meal intake prior to performance amongst elite players compared to lower level players, suggesting that elite level players may be more aware of the importance of CHO. Similarly, the second sub-analysis established that junior players opt for CHO dominant meals prior to performance more readily than adult players; however, no clear reasoning can be given alongside these findings. It is difficult to draw conclusions about playing standard and/or age and nutrition habit variance, due to the small and unbalanced sample sizes associated with this sub-analyses and lack of research in these fields.

In terms of meal specificity, foods regularly cited included pasta, oatmeal, and sandwiches which are all associated with high CHO content and energy provision [28]. A small number of players reported the importance of meal timing and digestibility alongside meal choice, referring to its impact on subsequent subjective gastrointestinal comfort and performance. Research supports the consumption of a pre-exercise meal, 3–4 h prior to sporting performance, to enable enhanced intestinal absorption and gastrointestinal tolerance [10,11]. With foods high in fat, protein and fibre associated with gastrointestinal issues, inclusive of bloating, stomach pain and indigestion [26], it is important that players adopt a pre-exercise nutritional regime promoting energy availability and gut tolerance [11].

With tennis performance demanding high energy availability, predominantly yielded from CHO oxidation, maintenance of blood glucose is important to spare muscle glycogen and attenuate the development of fatigue [29]. Results indicated that all players adopted a nutritional strategy during matches. Players cited water (94%), banana(s) (86%), and sports drink(s) (50%) consumption as the most commonly used nutritional aids (Figure 1). The consumption of CHO–electrolyte solid and liquid sources during exercise has been shown to elongate time to fatigue in several sporting domains [30,31]. However, tennis specific research relating to 6% CHO beverage ingestion and simulated tennis performance fails to support these findings, with no performance improvements established [32,33].

When asked whether players targeted a specific volume of fluid during a match, the majority (77%) of players stated that they did not. Of the 23% that did stipulate targeting a certain volume of fluid, 50% aimed for 500 mL^−1^ L, 6% between 1–2 L and 44% >2 L. Research to date investigating sweat rates and sodium losses following competitive tennis have reported sweat rates between 1.1 ± 0.4 L h^−1^ and 2.0 ± 0.5 L h^−1^ and sodium losses of 1.1 ± 0.4 g [34,35]. As tennis matches involve multiple breaks in play, the nature of the sport enables opportunities for players to take on fluids and electrolytes [2]. The present study indicated that only 50% of players typically consume sports drinks (containing CHOs and electrolytes) during matches. Consuming water alone during exercise can increase urine output alongside the rapid reduction in plasma sodium concentration, thus negatively impacting the body’s fluid balance [36,37]. With sodium balance linked with muscle functioning and, in extreme cases, hyponatremia [38] it is important that players adequately plan to manage fluid intake [39]. Results suggest that, currently, players are not doing enough to negate the negative effects associated with sodium and water misbalances.

Research has found an increased match length to significantly impact on the physiological demands of tennis performance, reducing indices of performance, including ground-stroke velocity and accuracy, and slowing racket-arm acceleration while serving [32]. The majority of players indicated utilising CHO rich nutritional aids (e.g., sports drinks, energy gels, sweets) to support performance during matches more than 2 h in duration. The most commonly cited non-nutrition-based performance aid was cold/wet towel use (39%). Although pre-cooling may provide some assistance for exercise performance in the heat [40], there is a lack of field-based evidence regarding the effect of this method on tennis performance [32,41,42].

When asked whether players adapt their eating habits when playing matches on different surfaces, inclusive of clay, hard, acrylic, tarmac and grass courts, only 14% of players reported doing so when playing on clay, stating that they eat more. No changes were stated when playing on any other court surface. Clay court matches are associated with longer rallies and greater playing times than all other courts. Consequently, matches on clay typically demand greater energy expenditure in comparison to other surfaces [43,44,45]. Altering nutrition habits to compensate for the increased energy demands would seem an appropriate recommendation for players. However, evidence from the current study would suggest the majority of players do not accommodate for this.

In the period after glycogen-lowering exercise, glycogen synthesis is a key priority for the previously contracted muscles [26]. When players were asked to consider their post-match nutrition, no clear trends were evident. Just 34% of players cited the consumption of CHO dominant meals immediately post-match. It has long been established that delayed CHO feeding can have a detrimental effect on muscle glycogen concentrations [46,47]. Tournament tennis often consists of multiple matches a day and consecutive days of matches [48]. Failure to maximise muscle glycogen stores prior to match-play may have a negative impact on performance, yet more research is required to substantiate this hypothesis.

When assessing nutrition habits the day after match-play, it was surprising to note that 39% of players cited the consumption of “nothing specific”. These findings suggest that a proportion of players do not tailor their diet for rest and recovery days. This is a concern, with strong evidence supporting the implementation of post-exercise nutritional strategies as a key means of improving the recovery of intramuscular glycogen [26], stimulating muscle protein synthesis (MPS) [11], attenuating reductions in muscle function [49], as well as enhancing overall adaptation to training and exercise [10]. With indices of technical and physical performance shown to reduce during tennis tournaments associated with fatigue [3,7], implementing effective strategies is important to improve players nutrition status and enhance future performances, as seen in other sports [50].

When asked whether recovery strategies were used to aid recuperation post-match play, the majority of players stated employing several strategies. Eighty percent reported using foam rollers, which has been previously shown to attenuate muscle soreness following a bout of physical activity [51,52]. However, there is currently little scientific evidence showing improved overall recovery benefits from its use [53,54]. Other cited methods included ice baths, protein shake intake, and hot baths. With bouts of high intensity exercise associated with appetite loss, liquid sources such as protein shakes can provide an alternative for players who cannot tolerate solid foods [39]. Additionally, as match start time and duration can be highly variable in tennis, planning high quality, nutrient-rich recovery meals can be difficult [2]. The consumption of 20–25 g of protein after exercise has been shown to stimulate MPS and lower the rate of muscle protein breakdown [55]. Thus, consuming protein shakes can be deemed as a viable nutrition strategy to enhance overall protein intake, to attenuate fatigue and facilitate recovery [9]. However, promoting a food first approach to meet this protein need is advocated [56], with high quality protein sources such as milk widely endorsed [57,58,59].

Ice baths/cold water immersion (classified by immersion in water <15 °C) have been shown to have a significant effect on reducing some biological markers of muscle damage and inflammation [60,61]. However, despite the large volume of research performed in this area, the effectiveness of its use is not clearly established [61]. Hot water immersion, classified by water temperatures >36 °C (i.e., hot baths) are a popular recovery strategy [62]. Yet the physiological effects of immersion in hot water remain to be elucidated [62].

When participants were asked why they employed recovery strategies, the primary influence was that of the coach/peers, followed by scientific literature (Table 1). At present, determining the best recovery strategy for tennis players is extremely challenging. With varied causes of fatigue (playing style, gender, training status, playing surface, ball type and environment) associated with tennis performance [5] and lack of tennis specific guidelines relating to recovery, there is a need for further research in this area.

It is prudent to note the inherent issues associated with questionnaire use and reporting dietary habits, with biases in relation to accuracy and precision often cited [63,64]. The response bias associated with self-reporting may have had an impact on the quality of data gained in the current study. Furthermore, the relatively small sample size makes generalising the findings to all tennis players difficult, with further research encouraged. That said, insights gained from the current study are important for players, coaches and practitioners alike, as well as giving governing bodies, such as the Lawn Tennis Association (LTA), a platform to support players development to maximise performance and recovery through education and further research in this field. Future research investigating the dietary habits of tennis players, including energy intakes and energy expenditure around training and competition, is recommended.

## 5. Conclusions

In summary, this study presents a novel insight into tennis players’ nutritional habits pre-, during and post-match-play as well as the recovery strategies used by players. Players appear to adopt varied eating habits on the days preceding and succeeding matches. Notably, the day of and during match-play appear to be when players take a more targeted approach towards their consumption, with some emphasis being shown towards energy provision (CHO and energy rich sources). However, it is evident that players’ performance nutrition requires improvement and that protocols adopted in this population remain highly varied. Further studies are needed to corroborate these findings, and future work should aim to identify the dietary habits of elite level players with the unique challenges associated with tennis competition and match-play.

## Figures and Tables

**Table 1:** Reasons for recovery strategy use, expressed as individual responses.

	Coach/Peer Influence	Easily Available	No Reason	Personal Preference	Saves Time	Scientific Literature
Creatine	2	0	0	0	0	3
Cherry Juice	0	0	0	0	0	1
Compression Garments	0	0	0	1	1	2
Foam Rolling	30	7	2	5	1	9
Hot Bath	2	4	0	9	0	3
Ice Bath	12	0	2	4	0	10
Protein Shake	5	3	1	9	0	8

## Supplementary Materials

The following are available online at http://www.mdpi.com/2072-6643/10/4/443/s1.
