# Supplementary material for: Investigating the Nutritional and Recovery Habits of Tennis Players
Source: Nutrients. 2018 Apr 3;10(4):443. doi: 10.3390/nu10040443 (PMC5946228; doi:10.3390/nu10040443)
Supplement: Supplementary file 1 [file nutrients-10-00443-s001.pdf]

Nutrition Habits Questionnaire

Please complete the following questionnaire honestly. Give as much detail as possible regarding types of foods eaten and stipulate what 'other' is, if and when it applies.

Are you?

Male ☐

Female ☐

How old are you?

What is your current playing standard?

County Level ☐

Regional Level ☐

Professional Level ☐

Please give further detail below (i.e. current club, rating, UTR and or ranking)

What do you typically eat and drink on the day before competition?

What does a typical pre-match meal (including fluids) consist of?

What do you typically eat and drink prior to a morning match (~9am start)?

What do you typically eat and drink on the day of a match (midday start)?

What do you typically eat and drink on the day of a match (afternoon ~2-3pm start)?

What do you typically eat and drink on the day of a match (evening ~6-7pm start)?

What do you typically eat or drink during a match?

Banana(s) ☐

Energy Gels ☐

Sugary Sweets (Candy) ☐

Chocolate ☐

Jaffa Cakes ☐

Water ☐

Sports Drinks (e.g. Lucozade sport) ☐

Juice/Squash ☐

Other… ☐

Please give details of the quantity consumed during a match, and the brand name, for the foods and or drinks selected in the previous question (e.g. 2 x SIS energy gels and 4 x McVities Jaffa cakes)

How do you gauge how much fluid to consume during a match?

By the number of games complete ☐

At the change of ends ☐

At the end of a set ☐

At the end of a match ☐

Time Passed ☐

Ad-libitum consumption according to thirst ☐

Other… ☐

Please provide reasons below for your choices

Do you specifically target a certain volume of fluid during a match?

Yes ☐

No ☐

If yes please specify quantity below. If no, please leave this question blank

500ml ☐

500ml – 1L ☐

>2L ☐

Other… ☐

What considerations do you make for a long match (>2 hours in duration)? Nutritional or otherwise?

Energy Gels ☐

Sports Drink ☐

Sugary Sweets (Candy) ☐

Cold/Wet Towel ☐

Other… ☐

Are there any specific recovery strategies you implement? Nutritional or otherwise?

Ice Bath ☐

Hot Bath ☐

Compression Garments ☐

Foam Rolling ☐

Protein Shakes ☐

Cherry Juice ☐

Creatine ☐

Other… ☐

Please choose the predominant reason for using the recovery strategies (if applicable).

	Scientific Literature	Peers, Coach, Friend Influence	Saves Time	Saves Money	Easily Available	Personal Preference
Ice Bath	☐	☐	☐	☐	☐	☐
Hot Bath	☐	☐	☐	☐	☐	☐
Compression Garments	☐	☐	☐	☐	☐	☐
Foam Rolling	☐	☐	☐	☐	☐	☐
Protein Shakes	☐	☐	☐	☐	☐	☐
Cherry Juice	☐	☐	☐	☐	☐	☐
Creatine	☐	☐	☐	☐	☐	☐
Other	☐	☐	☐	☐	☐	☐

What do you typically eat or drink immediately post-match for recovery (within 0-60 minutes)?

What do you typically eat or drink within three hours' post-match for recovery?

What do you typically eat or drink before bed post-match for recovery? (i.e., the same night as the match)

What do you typically eat or drink the day after a match for recovery?

Do you change your eating habits when playing on a clay court?

Yes ☐

No ☐

If yes, what do you change and why?

Do you change your eating habits when playing on a hard/tarmac/acrylic court?

Yes ☐

No ☐

If yes, what do you change and why?

Do you change your eating habits when playing on a grass court?

Yes ☐

No ☐

If yes, what do you change and why?

END

Thank you for taking the time to complete this questionnaire.
